# Single Stage Nipple-Sparing Mastectomy and Reduction Mastopexy in the Ptotic Breast

**DOI:** 10.1155/2018/9205805

**Published:** 2018-03-12

**Authors:** M. E. Pontell, N. Saad, A. Brown, M. Rose, R. Ashinoff, A. Saad

**Affiliations:** ^1^Department of Surgery, Drexel University College of Medicine, Philadelphia, PA, USA; ^2^Department of Surgery, University of Maryland, Baltimore, MD, USA; ^3^Department of Breast Surgery, Cancer Care Institute, Egg Harbor Township, NJ, USA; ^4^The Plastic Surgery Center, The Institute for Advanced Reconstruction, Egg Harbor Township, NJ, USA

## Abstract

**Purpose:**

Given the proposed increased risk of nipple-areolar complex (NAC) necrosis, nipple-sparing mastectomy (NSM) is generally not recommended for patients with large or significantly ptotic breasts. NAC preserving strategies in this subgroup include staged or simultaneous NSM and reduction mastopexy. We present a novel approach towards simultaneous NSM and reduction mastopexy in patients with large, ptotic breasts.

**Methods:**

Literature pertaining to NSM for women with large, ptotic breasts was reviewed and a surgical approach was designed to allow for simultaneous NSM and reduction mastopexy in such patients.

**Results:**

Eight patients underwent bilateral NSM with simultaneous reduction mammaplasty and immediate reconstruction. The majority of breasts demonstrated advanced ptosis (69% grade III, 31% grade II) and the average breast volume excised was 760 grams. In those patients without a history of smoking, NAC necrosis rates were 0%. In those patients with a history of smoking, 83% of breasts experienced NAC necrosis (60% total, 40% partial). One hundred percent of patients who smoked experienced some degree of NAC necrosis. Among breasts with grade II versus grade III ptosis, NAC necrosis rates were roughly equal.

**Conclusions:**

Historically, patients with large, ptotic breasts were excluded from NSM due to the proposed increased risk of NAC necrosis. This study demonstrates a safe approach towards NSM and reduction mastopexy using an inferior, wide-based, epithelialized pedicle. While all patients eventually achieved satisfactory results, there was an association between smoking and NAC necrosis. Smoking cessation is paramount to the operation's success.

## 1. Introduction

Nipple-sparing mastectomy (NSM) is a contemporary derivative of subcutaneous mastectomy (SCM), which was originally performed for fibrocystic disease of the breast [1, 2]. NSM is an increasingly popular alternative to skin-sparing mastectomy (SSM), as it allows for preservation of the nipple-areolar complex (NAC) [1, 3, 4]. With proper patient selection, NSM can be used in both the prophylactic and the therapeutic settings [1, 4–6]. Regardless of the indication, the central tenets of NSM are to remove the glandular breast tissue while maximizing structural preservation of the breast and adhering to oncologic standards [1, 3]. The trend towards the development of more advanced NSM modifications is driven by patient demand and an increasing amount of literature documenting its therapeutic success [7].

From an oncologic perspective, NSM is reserved for patients with tumors that do not involve the skin, are less than three centimeters in diameter, and are at least two centimeters away from the NAC [4, 8]. This procedure is a safe option for the treatment of breast carcinoma, and tumor recurrence rates are low [4, 5, 8–10]. Patients with excessively large and/or ptotic breasts or clinically palpable locoregional lymphadenopathy are generally excluded from therapeutic NSM [4, 8]. From a prophylactic standpoint, bilateral mastectomy remains a point of controversy and some surgeons do advocate for its use in high-risk patients who have a strong genetic predisposition towards developing breast cancer [10, 11]. On the other hand, contralateral prophylactic mastectomy (CPM) for risk reduction in patients with primary breast cancer is well supported [10, 11]. For appropriately selected patients, prophylactic mastectomy can reduce the risk of developing breast cancer by 80–95%, even in the presence of a retained NAC [4, 5, 10, 12]. As such, NSM is an important option in the prevention and treatment of breast cancer [12]. Additionally, NAC preservation has a positive impact on patient satisfaction [13, 14].

**Figure 1:**
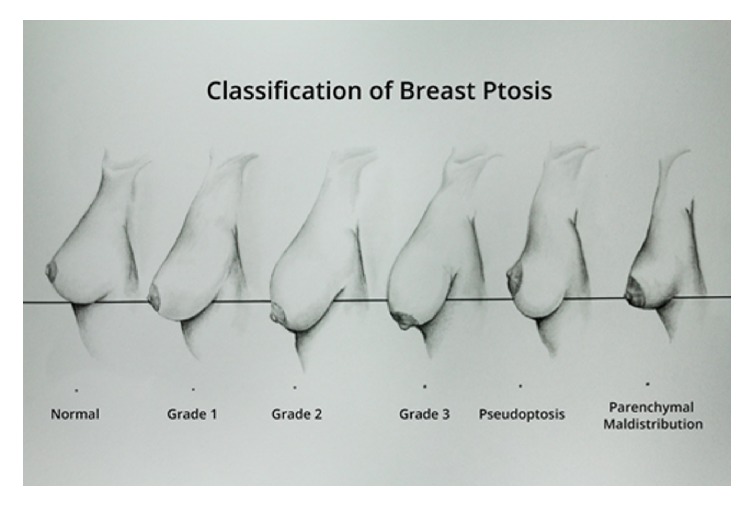
Artist's depiction of the breast ptosis grading system proposed by Regnault et al.* Normal*: areola above the inframammary fold (IMF) and above the gland contour;* Grade I*: areola at the IMF and above the gland contour;* Grade II*: areola below the IMF and above the gland contour;* Grade III*: areola below the IMF and below the gland contour;* Pseudoptosis*: areola at the IMF with glandular ptosis;* Parenchymal Maldistribution*: areola at the IMF with loose, hypoplastic glandular skin.

Nevertheless, the ability to perform a NSM can be restricted by patient anatomic factors. The procedure is generally not recommended for patients with breast volume exceeding 500 grams or grade II or III ptosis given the proposed increased risk of NAC necrosis (Figure 1) [4, 8]. Potential strategies for this patient subgroup include staged NSM and reduction mastopexy or NSM with simultaneous reduction mastopexy [1, 10, 13, 15–21]. Here, we present an alternate way to perform a simultaneous NSM and reduction mastopexy with breast reconstruction for females with large, ptotic breasts. This technique may provide a suitable option for such women who seek NAC preservation and wish to avoid multiple operations. Additionally, implant-based reconstruction may obviate a longer procedure for those who cannot tolerate a free-flap transposition.

## 2. Methods

A review of the literature was conducted on all cases of NSM and reduction mastopexy for women with large-volume, ptotic breasts. Based on the results, a modified surgical approach was created, designed to allow for simultaneous NSM and reduction mastopexy for women with high-grade ptosis and large-volume breasts. This study was conducted under the approval of the institutional review board of AtlantiCare Medical Center. All of the NSMs were performed by a single breast surgeon (AB) and the reconstructive procedures were performed by one, or occasionally two, of the plastic surgeons (AS, RA, and MR).

All mastectomies were nipple-sparing and were performed simultaneously with a reduction mammaplasty. Eight patients were included in this study, for a total of sixteen mastectomies (*n* = 16). Inclusion criteria consisted of patients with grade II or III breast ptosis who were candidates for prophylactic (five patients, ten breasts) or therapeutic (three patients, six breasts) NSM. After NSM and simultaneous reduction mammaplasty, patients underwent immediate placement of tissue expanders or reconstruction by deep inferior epigastric perforator (DIEP) flaps. In the tissue expander group, implants were inserted during the second-stage procedure. Additional minor revisions were made as necessary.

Data collection included patient demographics, preoperative indications, and active comorbid conditions at the time of surgery. All technical data, perioperative complications, and revision procedures were recorded and patients were followed up until all wounds had healed.

### 2.1. Surgical Technique

**Figure 2:**
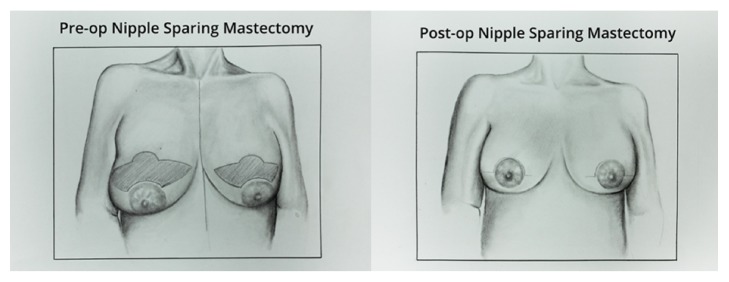
Artist's depiction of pre- and postoperative markings for simultaneous nipple-sparing mastectomy and reduction mastopexy. A “boomerang” shaped supra-areolar incision is made, through which breast tissue and a variable amount of skin are excised. The edges are reapproximated after insertion of a tissue expander.

Nipple-sparing mastectomy with this technique involved a supra-areolar incision with lateral and medial extensions (Figure 2). Retroareolar breast tissue was sent for frozen section to rule out carcinoma involvement of the NAC and thin mastectomy flaps were raised superiorly and inferiorly with the NAC being thus carried on a broad, inferior-based epithelialized dermal pedicle. A variable amount of skin above the supra-areolar incision was excised in a pattern akin to a boomerang, with the width of the boomerang adjusted based upon how much lift was needed to bring the NAC into a more normal anatomic position. After raising the skin flaps up to the level of the clavicle superiorly, the inframammary fold inferiorly, the sternal border medially, and the anterior edge of the latissimus muscle laterally, the breast tissue was sharply dissected off of the pectoralis major muscle.

At this point, if expanders were used, the pectoralis major muscle was lifted off of the chest wall sharply to allow for a submuscular pocket to cover the superior and superior-medial portions of the expander. Various acellular-dermal matrix (ADM) products were utilized to create the inferior and inferolateral coverage over the expander. Expander size was chosen based upon base width of the native breast and other chest-wall measurements. The ADM was sutured into place along the inframammary fold, the lower border of the pectoralis muscle, and the lateral chest wall with 2-0 Vicryl sutures. The expander was placed and a drain was placed below the skin but above the expander pocket. All expanders were partially inflated with sterile saline and the SPY Intraoperative Perfusion Assessment System (distributed in North America by LifeCell Corp., Branchburg, NJ; manufactured by Novadaq Technologies Inc., Richmond, British Columbia, Canada) was used at this point to confirm NAC and mastectomy flap viability. Closure consisted of two layers of 3-0 and 4-0 monocryl followed by Dermabond.

If a DIEP flap was used, then a two-team approach was used with one team member dissecting out the recipient vessels in the chest while a second team member was raising and dissecting out the DIEP flap on the abdominal wall. Coupled venous anastomoses were used in all cases and hand-sewn arterial anastomoses were used in all cases with 8-0 nylon sutures. Flaps were stabilized onto the chest wall with 3-0 Vicryl sutures after restoration of blood flow. Abdominal fascia was repaired with 1-0 PDS sutures and the abdominal flap was closed with 0-0 PDS for the fascial layer, 3-0 monocryl for the dermal layer, and 4-0 monocryl for the skin. Ten-millimeter flat channel drains were used in the abdomen and behind the DIEP flaps in all cases. Flaps were monitored with Doppler ultrasound and clinical exam every fifteen minutes for 3 hours and then hourly thereafter.

## 3. Results

Eight patients underwent bilateral NSM with simultaneous reduction mammaplasty and breast reconstruction. A total of sixteen mastectomies were performed. Average age was 49 years, 75% of patients had comorbid conditions, and 63% of patients were actively smoking at the time of surgery. Five patients met criteria for prophylactic resection and three patients met criteria for therapeutic resection. Sixty-nine percent of breasts demonstrated grade III ptosis and the remainder were grade II. Seventy-five percent of patients had bilateral nipple-sparing mastectomies with immediate reconstruction with a tissue expander and implant insertion on a later date. The remaining 25% of patients underwent immediate reconstruction with DIEP flap. Average volume of breast tissue excised was 760 grams. In the tissue expander group, the average expander size was 560 cc with average initial expander volumes of 240 cc (Table 1). SPY intraoperative perfusion confirmed viable mastectomy flaps and nipple-areolar complexes.

There were a total of 11 mastectomies that were not complicated by NAC necrosis. One patient developed unilateral hematoma. The average age in this group was 49 years, one patient was actively smoking at the time of surgery, and 91% of patients had active comorbid diseases. Twenty-seven percent of procedures were therapeutic, and 73% were prophylactic. Breast ptosis grades were 81% grade III and 19% grade II. Eighty-one percent of mastectomies were reconstructed initially with tissue expanders and 19% underwent immediate DIEP flap reconstruction. In those patients who were nonsmokers, NAC necrosis rates were 0% (Table 2).

There were a total of five mastectomies that were complicated by NAC necrosis (60% total, 40% partial). Of these, two breasts also developed seromas and one developed mastectomy flap necrosis. Average age in the NAC necrosis group was 59 years. All patients who developed NAC necrosis were smokers and only one patient had active comorbidities. Forty percent of procedures were prophylactic and 60% were therapeutic. Ptosis grades were 40% grade III and 60% grade II. Sixty percent of patients in this group underwent reconstruction by a tissue expander and the remaining 40% underwent immediate DIEP flap-based reconstruction (Table 3).

**Figure 3:**
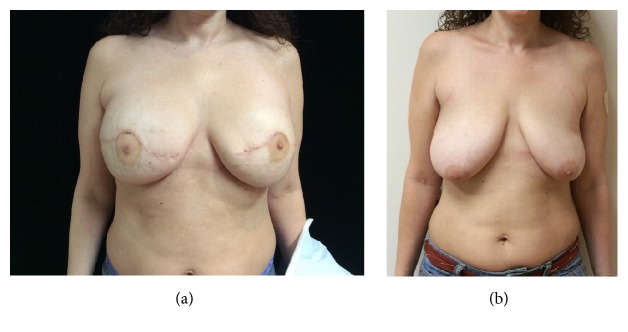
Pre- (a) and postoperative (b) photographs after simultaneous nipple-sparing mastectomy and reduction mastopexy with implant-based reconstruction.

Rates of partial and total NAC necrosis rates were 12.5% and 18.7%, respectively. Comparison of the breasts that experienced NAC necrosis with those that did not revealed average ages of 59 and 49 years, respectively. One hundred percent of patients who experienced NAC necrosis were smokers versus 9% in the NAC intact group. Twenty percent of cases of NAC necrosis had associated comorbidities versus 91% in the NAC intact group. On average, the percentage of therapeutic mastectomies was slightly higher in the NAC necrosis group; however, the percentage of grade III ptosis was lower. Reconstruction methods were similar in both groups (Table 4). All patients were eventually able to heal their incisions and postoperative wounds (Figure 3).

## 4. Discussion

Female patients with large-volume, severely ptotic breasts who are candidates for NSM pose a specific challenge to reconstructive surgeons. Most surgeons are reluctant to perform a simultaneous NSM and reduction mastopexy given the supposed increased risk of NAC and skin flap necrosis [1, 4, 7, 12, 13, 22]. Some authors argue that advanced breast ptosis may further contribute to the development of this complication and may also impair NAC repositioning and management of the skin envelope when necessary [1, 4, 7, 12, 22]. Studies propose that high-grade ptosis and/or excessive breast volume may increase the length of the skin flap required to supply the NAC, thereby compromising vascular supply [22]. Additionally, some argue that substantial amounts of breast tissue need to be left behind to ensure NAC and flap perfusion resulting in an inadequate mastectomy [13]. Nevertheless, several studies have reported options for women with large breasts and/or advanced ptosis who meet criteria for NSM [1, 13]. These techniques can be broadly subdivided into staged NSM and reduction mastopexy [1, 13, 23] or simultaneous reduction mastopexy with NSM [10, 16–21].

Review of the literature revealed three studies that focused on staged reduction mastopexy and NSM for women with large, ptotic breasts [1, 13, 23]. Spear et al. published a series of cases in which such patients were offered staged NSM and reduction mastopexy [13]. Partial NAC necrosis rates were roughly 12.5% and there were no cases of total NAC necrosis [13]. While results were promising, the authors felt this procedure would be best suited for patients with medium-volume breasts with moderate ptosis [13]. Two other studies employed the use of immediate flap-based reconstruction after NSM with a delayed reduction mastopexy [1, 23]. These studies used various free flaps to support NAC perfusion after NSM, and reduction mastopexy for moderately to severely ptotic breasts was performed on a later date [1, 23]. The main disadvantage in the staged approach is that the patient requires two major surgeries. Additionally, patients with active comorbid conditions may not be able to tolerate a lengthy free-flap procedure. The mean percentages of partial and total NAC necrosis in the staged group were 4.16% and 1%, respectively (Table 5).

In the 1970s, several studies examined the utility of SCM with NAC preservation with simultaneous reduction mastopexy for patients with large breasts and/or severe ptosis [16–19, 21]. While these studies showed promising results regarding NAC preservation, several failed to specify the breast size or degree of ptosis [16, 19]. Additionally, early studies focused on SCM with NAC preservation, which likely resulted in a less comprehensive mastectomy as indications at that time were strictly prophylactic. The literature suggests that breast tissue quantities now considered unacceptable for conventional NSM were left behind during SCM to support NAC and flap perfusion [1]. Nevertheless, there has been a resurgence of interest in simultaneous NSM and reduction mastopexy, likely reflecting the increase in patient demand [7]. Two studies in 2010 and 2011 demonstrated good results regarding NAC preservation; however, the data reported did not allow any conclusions to be drawn regarding the breast size or degree of ptosis [7, 24]. Al-Mufarrej et al. and Rivolin et al. reported on two series of patients with medium- to large-volume breasts and moderate ptosis who underwent NSM with simultaneous reduction mastopexy [10, 15]. Results of these studies demonstrated excellent NAC preservation in prophylactic and therapeutic scenarios; however, the issue of severe breast ptosis did not appear to be addressed [10, 15]. Average partial and total NAC necrosis rates in the simultaneous group were 3.65% and 2.16%, respectively (Table 5).

**Figure 4:**
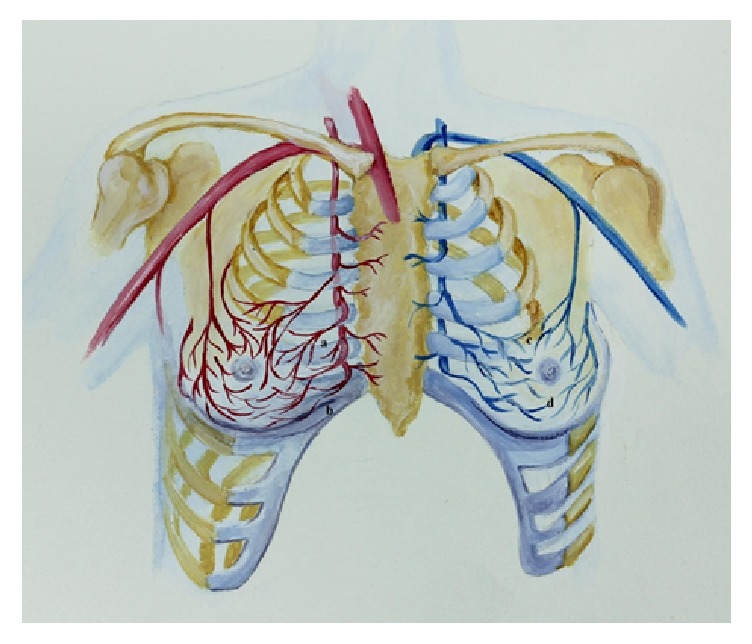
Artist's depiction of the arterial supply* (right breast)* and venous drainage* (left breast)* to the nipple-areolar complex (NAC). The most important contributor to NAC perfusion arises from the third internal thoracic artery perforator (a). This branch travels medially from its origin and courses just under the NAC where it gives off tributaries to the periareolar network. The anterior intercostal arteries originate more inferiorly and course along the inframammary fold before giving their contributions to the arterial supply of the NAC (b). The NAC is drained through superior (c) and inferior (d) horizontal venous slings that ultimately drain into the thoracic and subclavian veins [17, 19].

The pedicled flap used in this study is a wide-based, epithelialized version of the traditional inferiorly based flaps used during reduction mammaplasty. The base of the flap was widened in attempt to preserve the natural arterial and venous supply to the NAC. The NAC receives arterial perfusion from a periareolar network that is supplied by perforating branches of the internal thoracic artery, the anterior intercostal arteries, and the lateral thoracic artery [25]. The most important contribution arises from the third internal thoracic artery perforator [25]. All of these arterial networks course towards the NAC in a medio- or lateroinferior direction (Figure 4). After formation of the periareolar plexus, the cutaneous perforators travel within the subcutaneous tissue before reaching the NAC and after mastectomy the NAC relies solely on these cutaneous branches as the underlying breast tissue has been removed [1, 26]. With respect to vascular outflow, the NAC is drained through a superior and inferior horizontal venous sling (Figure 4) [27]. After mastectomy, the NAC drainage relies heavily on the superficial, inferiorly coursing venous network [27]. The cutaneous venous system is even more superficial than the arterial network and as such is more likely to be damaged during deepithelialization [27]. Necrosis of the NAC results from either arterial or venous insufficiency and the latter appears to be even more prevalent with larger breast resection volumes [13, 27–29]. Given the vascular anatomy of the NAC, expanding the base of the pedicle in a lateral fashion should theoretically preserve more of the arterial supply and venous drainage. In addition, by maintaining an epithelialized pedicle, the cutaneous vascular perforators that nourish the NAC should also be better preserved. The importance of vascular preservation is amplified with larger breast volumes [27, 28]. In theory, such dissection should offer anatomical advantages when compared to other techniques that use narrow, deepithelialized pedicles to support NAC perfusion after NSM and reduction mammaplasty.

Limitations of this study include the small sample size and thus an inability to draw statistically significant conclusions. In addition, while the average breast volume excised was 760 grams, several of the patients did not have excised breast volumes recorded and therefore NAC necrosis could not be analyzed alongside breast volumes. Overall rates of partial and total NAC necrosis were 12.5% and 18.7%, respectively. The discordance between SPY perfusion results and NAC survival may represent either a lack of diagnostic accuracy on behalf of the SPY system or, more likely, the complex microvascular disease that develops in active smokers. While these complication rates do appear high, subset analysis reveals that all patients who had NAC necrosis were smokers and all patients who smoked developed NAC necrosis. Excluding the subset of patients who smoked, partial and total NAC necrosis rates were 0%. Such complications did not appear to be related to patient age, the presence or absence of comorbidities, indication for procedure, grade II versus III ptosis, or the type of reconstruction performed.

In summary, this study presents an alternate technique for simultaneous NSM and reduction mastopexy for women with large, ptotic breasts. Using this method, comparable amounts of NAC preservation were able to be achieved in what has historically been considered a high-risk patient group for this procedure. While NSM has traditionally been avoided in this patient subgroup, this study supports its inclusion when considering a patient for either prophylactic or therapeutic NSM. Using a wide, inferior, epithelialized pedicle based on the vascular anatomy of the NAC, comparable rates of NAC preservation are possible, even in patients with large-volume, severely ptotic breasts. Options for immediate reconstruction exist, and a staged approach may not be necessary. Emphasis on smoking cessation is paramount to the success of the operation.

## Figures and Tables

**Table 1:** Patient characteristics and procedural specifics.

Pt	Age	Sex	Smoker	PMH	Indication	Ptosis grade	Technique	Expander size	Breast volume excised (R/L)
1	55	F	No	Hypertension	Biopsy with atypical cells in the setting of bilateral silicone injections	III (B/L)	Bilateral NSM with reduction mammaplasty and expander insertion	350 cc	665 gr/740 gr

2	30	F	No	Asthma, depression	BRCA mutation	III (B/L)	Bilateral NSM with reduction mammaplasty and expander insertion	800 cc	1240 gr/1316 gr

3	54	F	No	Gastric cancer, thyroid disease, peripheral neuropathy	BRCA mutation	III (B/L)	Bilateral NSM with reduction mammaplasty and expander insertion	400 cc	429 gr/449 gr

4	58	F	No	Thyroid disease	BRCA mutation	III (B/L)	Bilateral NSM with reduction mammaplasty and expander insertion	800 cc	1006 gr/776 gr

5	52	F	Yes	None	Unilateral, multifocal DCIS	III (B/L)	Bilateral NSM with reduction mammaplasty and DIEP flap reconstruction	N/A	NR

6	58	F	No	Hypertension, diabetes mellitus	Unilateral invasive breast cancer, BRCA	III/II (R/L)	Bilateral NSM with reduction mammaplasty and DIEP flap reconstruction	N/A	546 gr/436 gr

7	32	F	Yes	None	Unilateral invasive breast cancer	II (B/L)	Bilateral NSM with reduction mammaplasty and expander insertion	500 cc	NR

8	55	F	Yes	Ovarian cancer, thyroid disease	BRCA	II (B/L)	Bilateral NSM with reduction mammaplasty and expander insertion	500 cc	NR

PMH: past medical history; R: right; L: left; B/L: bilateral; NSM: nipple-sparing mastectomy; BRCA: breast cancer susceptibility gene; DCIS: ductal carcinoma in situ; N/A: not applicable; DIEP: deep inferior epigastric perforator; NR: not reported.

**Table 2:** Breasts that did not experience NAC necrosis stratified by individual mastectomy.

Pt.	NAC necrosis	Wound complications	Age	Smoker	PMH	Indication	Ptosis grade	Reconstruction
1 (R)	No	Hematoma	55	N	Y	Prophylactic	III	Expander
1 (L)	No	None	55	N	Y	Prophylactic	III	Expander
2 (R)	No	None	30	N	Y	Prophylactic	III	Expander
2 (L)	No	None	30	N	Y	Prophylactic	III	Expander
3 (R)	No	None	54	N	Y	Prophylactic	III	Expander
3 (L)	No	None	54	N	Y	Prophylactic	III	Expander
4 (R)	No	None	58	N	Y	Prophylactic	III	Expander
4 (L)	No	None	58	N	Y	Prophylactic	III	Expander
6 (L)	No	None	58	N	Y	Therapeutic	II	DIEP
6 (R)	No	None	58	N	Y	Therapeutic	III	DIEP
7 (L)	No	None	32	Y	N	Therapeutic	II	Expander

Pt.: patient number; NAC: nipple-areolar complex; PMH: past medical history; N: no; Y: yes; R: right breast; L: left breast; DIEP: deep inferior epigastric perforator.

**Table 3:** Breasts that experienced NAC necrosis stratified by individual mastectomy.

Pt.	NAC necrosis	Wound complications	Age	Smoker	PMH	Indication	Ptosis grade	Reconstruction
5 (R)	Partial	None	52	Y	N	Therapeutic	III	DIEP
5 (L)	Total	Flap necrosis	52	Y	N	Therapeutic	III	DIEP
7 (R)	Partial	None	32	Y	N	Therapeutic	II	Expander
8 (R)	Total	Seroma	55	Y	Y	Prophylactic	II	Expander
8 (L)	Total	Seroma	55	Y	Y	Prophylactic	II	Expander

Pt.: patient number; NAC: nipple-areolar complex; R: right breast; L: left breast; Y: yes; N: no; DIEP: deep inferior epigastric perforator.

**Table 4:** Breasts that experienced NAC necrosis compared to those that did not.

Group	Number of breasts	Avg. age	Smokers	Comorbidities present	Therapeutic versus prophylactic	Ptosis grade (II versus III)	Expander versus DIEP
NAC necrosis	5	59 years	100%	20%	60% versus 40%	60% versus 40%	60% versus 40%
NAC intact	11	49 years	9%	91%	27% versus 73%	19% versus 81%	81% versus 19%

NAC: nipple-areolar complex; Avg.: average; DIEP: deep inferior epigastric perforator.

**Table 5:** Table reviewing all of the studies published on nipple-sparing mastectomy in large-volume, ptotic breasts from 1970 to 2016.

	Technique	Reconstruction	Sample size (number of breasts)	Indication	Ptosis/breast volume	Partial NAC necrosis	Total NAC necrosis	Other complications
*Simultaneous mastopexy and NSM*								

Goulian & McDivitt, 1972	SCM with reduction mastopexy	Implant None	24	Risk reduction	Medium-large Not specified	None	None	Hematoma (NR)

Biggs et al., 1977	SCM with reduction mastopexy	Implant	33	NR	Not specified Min-Mod ptosis	None	None	Partial flap necrosis (1) Capsular contractures (8) Atrophy requiring excision (1) Explant (1)

Jarrett et al., 1978	SCM, reduction mastopexy, and free nipple graft	Implant	44	Risk reduction	Large volume Severe ptosis	None	None	NR

Gibson, 1979	SCM with reduction mastopexy	NR	NR	Risk reduction	Not specified	None	None	NR

Rusby & Gui, 2010	NSM with reduction mastopexy	Expander	16	Risk reduction	NR NR	NR	6.3%	None

Nava et al., 2011	NSM with reduction mastopexy	Implant	13	Therapeutic	NR NR	NR^#^	NR^#^	NR^#^

Rivolin et al., 2012	NSM with periareolar pexy	Implant	22	Therapeutic	Medium-large volume Moderate ptosis	13.6%	4.6%^*∗*^	None

Al-Mufarrej et al., 2013	NSM with reduction mastopexy	Expander Implant	48	Risk reduction	Large volume Moderate	8.3%	4.2%	Infected implant (2.1%) Implant rupture (14.6%) Hematoma (2.1%) Capsular contracture (4.2%)

Pontell et al., 2016 (this report)	NSM with reduction mastopexy	Expander DIEP Flap	16	Risk reduction Therapeutic	Large volume Grade II/III ptosis	0%^*∗∗*^	0%^*∗∗*^	Hematoma Seroma Mastectomy flap necrosis

*Staged mastopexy and NSM*								

Schneider et al., 2012	NSM with immediate flap placement and staged reduction mastopexy	TUG Flap DIEP Flap	34	NR	Large volume Grade II/III ptosis	None	3%	Hematoma (3%)

DellaCroce et al., 2015	NSM with immediate flap placement and staged reduction mastopexy	DIEP Flap SGAP Flap	110	Risk reduction Therapeutic	Medium-large volume Grade II/III ptosis	None	None	Partial mastectomy flap necrosis (3.6%) Incisional dehiscence (8%) Hematoma (2.7%) Partial flap necrosis (1.8%)

Spear et al., 2012	Reduction mastopexy followed by NSM	Implant Tissue expander	24	Risk reduction Therapeutic	Medium volume Grade II/III ptosis	12.5%	None	Breast infection (8%) Skin flap necrosis (17%) Explant (4%)

NAC: nipple-areolar complex; NSM: nipple-sparing mastectomy; SCM: subcutaneous mastectomy; NR: not reported; DIEP: deep inferior epigastric perforator; TUG: transverse upper gracilis; SGAP: superior gluteal artery perforator. ^#^Complications were not stratified by NSM (SRM) versus SSM status. ^*∗*^This study mentions the exclusion of one patient who had total NAC necrosis. ^*∗∗*^These rates exclude the patients who were smokers, including patients with partial and total NAC necrosis rates of 12.5% and 18.7%, respectively.
